# Osteopontin and the C-terminal peptide of thrombospondin-4 compete for CD44 binding and have opposite effects on CD133+ cell colony formation

**DOI:** 10.1186/1756-0500-2-215

**Published:** 2009-10-23

**Authors:** Gulzhakhan Sadvakassova, Monica C Dobocan, Luis F Congote

**Affiliations:** 1 Endocrine Laboratory, McGill University Health Centre, 687 avenue des pins, ouest, Montreal, Canada H3A 1A1

## Abstract

**Background:**

C21, the C-terminal peptide of thrombospondin-4, has growth promoting activity and was discovered as one of several erythropoietin-dependent endothelial proteins. C21 stimulates red cell formation in anemic mice and is a growth factor for CD34+ and CD36+ hematopoietic cells, skin fibroblasts and kidney epithelial cells. ROD1 has been identified as an intracellular mediator. Nothing is known about the existence of putative C21 receptors on plasma membranes of target cells.

**Findings:**

We analyzed the nature of C21-binding proteins in cell lysates of skin fibroblasts using C21 affinity columns. The membrane receptor CD44 was identified as C21-binding protein by mass spectrometry. We were unable to demonstrate any direct involvement of CD44 on cell growth or the effect of C21 on cell proliferation. A soluble form of CD44 was synthesized in insect cells and purified from culture supernatants with a combination of PVDF filtration in the presence of ammonium sulphate and HPLC. Both osteopontin and hyaluronic acid competitively displaced Biotin-C21 binding to CD44. In a colony-forming assay using primitive CD133+ hematopoietic stem cells from cord blood, osteopontin and C21 had opposite effects and C21 reduced the inhibitory action of osteopontin.

**Conclusion:**

CD44 is a C21-binding membrane protein. We could not demonstrate an involvement of CD44 in the proliferative action of C21. Nevertheless, based on the antagonism of C21 and osteopontin in hematopoietic precursors, we speculate that C21 could indirectly have a major impact on hematopoietic stem cell proliferation, by hindering osteopontin membrane binding at the level of the bone marrow niche.

## Background

Erythropoietin (EPO) is the most important cytokine involved in the production of red cells [1]. In endothelial cells, EPO stimulates the synthesis of proteins involved in red cell formation, such as thrombospondins (TSPs) 1 and 4 [2,3]. TSPs are very large extracellular matrix glycoproteins with multiple functions [4]. The biological activity of the C-terminal, amphipathic peptide of TSP-4 (C21) has been described only recently [2] and therefore nothing is known about putative membrane receptors for the peptide on the surface of target cells. Our results on the search for potential C21 receptors can be summarized in three points: (1) CD44 was identified as a C21-binding membrane protein in lysates of skin fibroblasts, but we were not able to demonstrate a direct involvement of CD44 in the mitogenic action of C21. (2) Binding studies with a recombinant CD44 indicated that osteopontin (OPN) competed with C21 for CD44 binding, suggesting a possible function of C21 as OPN antagonist. (3) C21 and OPN had opposite effects on colony formation in cultures of primitive hematopoietic stem cells. Therefore, we speculate that C21 binding to CD44 could indirectly stimulate hematopoietic stem cell proliferation by preventing the inhibitory action of osteopontin (OPN).

## Methods

Cell cultures of human skin fibroblasts, 293T kidney epithelial cells and Trichoplusia ni insect cells (TN) were done as previously described [5,6]. Cord blood CD133+ precursor cells (Lonza) were cultured in methylcellulose medium (Methocult H4536, Stem Cell Technologies) supplemented with 3 U/ml EPO in the presence of 1 μM C21 [5] or 0.5 μg/ml recombinant human osteopontin (OPN, carrier-free, R&D Systems). The cells (5000/ml) were plated on 12-well plates (Costar, 0.5 ml/well). The plasmid pENTR containing the cDNA coding for transcript 4 of human CD44, CD44v4 (Ultimate ORF Clone Collection, ID IOH53593, Invitrogen) was modified to introduce a stop codon at the beginning of the extracellular section of the transmembrane domain of CD44 with the "QuikChange^®^" Site directed mutagenesis kit (Stratagene). The modified cDNA was integrated into the destination vector pIB/V5-His-DEST with clonase (Invitrogen Gateway™ technology) for insect cell production and the complete CD44 sequence was integrated using the same method into the pLenti6/V5-DEST plasmid for lentiviral transduction in 293T cells.

C21-affinity chromatography, gel electrophoresis and MS analysis were done as previously described [5].

Soluble CD44 was isolated from three day supernatants of cultures from transfected TN cells cultured in serum-free Excel 405 medium (Sigma). 100 ml of ice-cold medium were mixed with 2 M Tris-HCl, pH 8 to a final Tris concentration of 0.1 M and stirred for 10 min. The precipitated proteins were separated by centrifugation at 10,000 × g for 20 min. The supernatant was mixed with 6 g SM-2 Adsorbent beads (Bio-Rad) and further stirred on ice for 20 min. The beads were trapped with 4 layers of cheese cloth and the remaining medium was mixed with 43 g ammonium sulfate and stirred for 1 h. The precipitated proteins were eliminated by centrifugation (20 min, 10,000 × g) and the supernatant was filtered through Durapore PVDF membranes (0.22 μm, Cat. SCGVU02RE, Millipore). The filter was cut in pieces, gently shook on a nutator at 8°C for 20 min, first with 5 ml 0.3% (w/v) Zwittergent 3-16 (Calbiochem) and then with 5 ml 1% (v/v) Triflouroacetic acid (TFA). The two extracts were mixed, warmed up to room temperature and applied to a Vydac semi-preparative C4 column (10 × 250 mm, Cat 214TP1010, Grace/Vydac). The column was washed with 30 ml 0.1%TFA and the extract components separated with a gradient of acetonitrile [7]. The collected fractions were immediately lyophilized, dissolved in 60 μl of the storage buffer [5]. The fractions containing CD44 were identified by applying 2 μl on nitrocellulose paper. The dot blots were developed, scanned and measured as previously indicated [8]. We used a monoclonal anti-CD44 antibody recognizing all CD44 isoforms (R&D Systems). Digestion of CD44 with peptide:N-glycosidase F (PNGase F, New England Biolabs) was done following the instructions of the manufacturer. Binding of biotinylated C21 (Biotin-C21) to CD44 on nitrocellulose membranes was done as previously described for ROD1 [5]. Binding competition studies were done with the same OPN preparation utilized for cell culture as indicated above and with human umbilical cord hyaluronic acid (potassium salt, Calbiochem).

## Results and discussion

### Identification and potential significance of CD44 as a C21 binding protein

**Figure 1:**
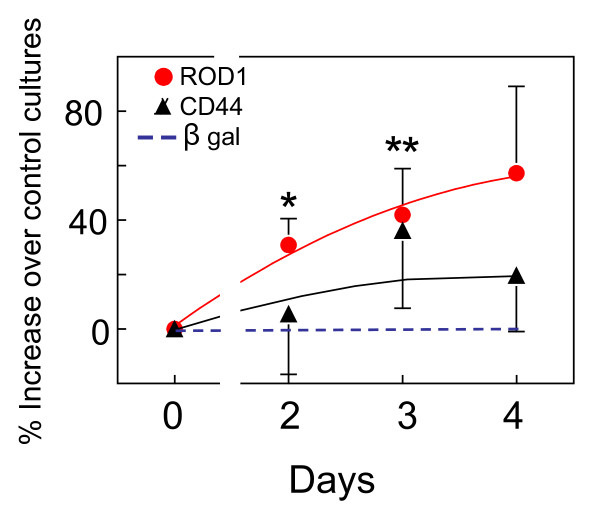
**Effects of CD44 on insect cell proliferation**. TN insect cells were permanently transfected with ROD1 [5] or CD44 as indicated in Methods and their growth in culture was compared with parallel control cell cultures transfected with β-galactosidase. The increase in cell numbers observed in ROD1 or CD44 transfected cultures was expressed as percentage of the number of cells observed in β-galactosidase transfected cells after the same day in culture. The values are mean ± SEM, from 5 to 6 experiments. Only the effects of ROD1 were statistically different from the controls (Krustal-Wallis and Dunn's multiple comparisons test). *, P < 0.05. **, P < 0.01.

CD44 is the only C21-binding cell membrane protein identified so far by mass spectrometry using the C21 affinity chromatography described in Methods. The Mascot (Matrix Science) search program indicated a match to CD44E [GenBank:CAA38951]. We found that another C21-binding protein, the nuclear protein ROD1, plays an active role on the mitogenic activity of C21 [5]. Our original hypothesis was that C21 binds first to CD44, is internalized (as has been demonstrated with the CD44 ligand hyalurodan [9]), goes to the nucleus and binds regulatory proteins such as ROD1, resulting in an inhibition of differentiation and/or stimulation of cell proliferation. ROD1 overexpression in skin fibroblasts and 293T cells resulted in an increased basal cell proliferation and in an enhancement of the mitogenic activity of C21 [5]. Insect cells stably transfected with ROD1 grew faster than non transfected or β-galactosidase-expressing cells (Fig 1). This is not surprising, because human ROD1 is active in lower eukaryotes such as yeast [10]. Although CD44-like, hyaluronic acid-binding proteins have been identified in primitive invertebrates [11], proliferation of stably CD44 transfected insect cells was not significantly different from that observed in β-galactosidase-transfected cells (Figure 1). The same was the case with 293T cells transduced with the complete CD44 sequence. We could not see any significant changes in basal- or C21-mediated increase of cell proliferation (results not shown). The problem with overexpression experiments is that negative results are inconclusive. This is particularly valid in the case of CD44. We are overexpressing only one of the many isoforms produced by alternative splicing [12] and a negative result with only one of the transcripts can not exclude the possibility that other CD44 molecules may be active.

### Biotin-C21 binding to CD44 is inhibited by hyaluronic acid and osteopontin

If CD44 is not implicated in the action of C21, it is still possible that C21-CD44 interactions may have an effect in the function of the membrane receptor in its capacity as a cell-adhesion molecule. The best known ligand of CD44 is hyaluronic acid (HA, hyaluronan), a ubiquitous cell matrix component, which plays an important role in hematopoiesis [13]. Of particular importance is the HA of the endosteal bone marrow niche, which plays a role in primitive hematopoietic stem cell proliferation and homing of transplanted cells to the marrow [14,15]. OPN, another CD44 ligand, is a negative regulatory protein of the hematopoietic stem cell niche [16,17].

**Figure 2:**
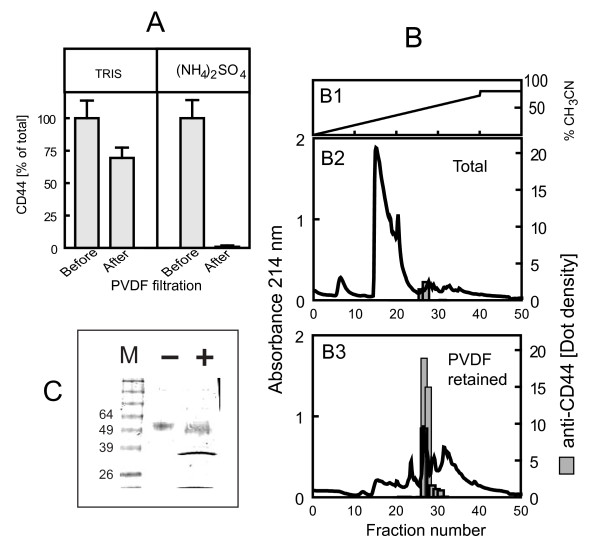
**Preparation of a soluble form of CD44 from insect cell culture medium**. **(A) **Ammonium sulfate is responsible for retention of soluble CD44 on PVDF filters. 22 μg CD44 prepared as indicated in Methods were dissolved in 0.3 ml 0.1 M Tris-HCl, pH8 and filtered through Millex-GV low protein binding Durapore, 0.22 μm pore size (Millipore SLGV033RS) in Tris buffer alone or with 3 M ammonium sulfate. The ultrafiltrates of ammonium-sulfate containing solutions were practically free of immunoreactive CD44, measured as indicated in Methods. Values are mean ± SEM (n = 4). **(B) **Reverse phase HPLC of the soluble ammonium sulfate fraction of cell culture supernatants. The supernatants of TN cultures expressing CD44 were processed as indicated in Methods and applied to a Vydac C4 semi-preparative column. **(B1) **Acetonitrile gradient, 0% to 72% (v/v) acetonitrile in 0.1% (v/v) TFA for 50 min, 2 ml/min. **(B2) **After centrifugation of the proteins precipitated with ammonium sulfate, the supernatant was desalted and concentrated by ultrafiltration (Centricon-20, cut-off 10 kDa, Millipore) and applied to the column. **(B3) **Instead of ultrafiltration, the supernatant was filtered through PVDF membranes (type GV, 0.22 μm). The proteins on the filter were extracted and applied to the column. The gray bars represent the density of the immunoreactive fractions using an anti-CD44 antibody. **(C) **PNGase digestion of recombinant soluble CD44. 120 ng CD44 were applied onto PAGE-SDS gels (10% acrylamide) and separated by electrophoresis before (-) and after (+) 1 h digestion with PNGase F. M = molecular weight markers. The gels were stained with coomassie blue.

Therefore, it was important to investigate the possible interference of C21 with binding to these two CD44 ligands, because it could indirectly play a role in homing and proliferation. For these purpose, we designed a soluble CD44 molecule from a CD44v4 cDNA as indicated in Methods and expressed the recombinant protein using insect cells. The method utilized solved a major technical problem of recombinant protein purification from cell culture supernatants: elimination of xenobiotic components, found in yeast-extract-based serum-free media utilized for protein production with insect or mammalian cells. For the first purification step, we utilized ammonium sulfate precipitation [18]. The human recombinant CD44 produced in insect cells could not be precipitated with ammonium sulfate. Nevertheless, we found that if a CD44 solution is filtered through PVDF membranes in the presence of ammonium sulfate, there is complete retention on the membranes (Figure 2A). This was not observed in the absence of ammonium sulfate. Retention without precipitation could be attributed to a Hofmeister ion effect [19,20] Figure 2B shows the importance of the utilization of PVDF for the retention of CD44. If supernatants are desalted by ultrafiltration, the vast majority of the culture contaminants are retained and CD44 recovery is low (Figure 2B). However, if PVDF retained material is solubilized and applied to HPLC columns, the vast majority of the contaminants of Figure 2B is eliminated and the recovery of CD44 is high (Fig 2C). MS analysis of the protein indicated that it corresponded to human CD44R1, [GenBank:CAA40133], a hematopoietic cell isoform of CD44 [21]. Treatment with a recombinant glycosidase indicated that the protein was extensively N-glycosylated by insect cells (Figure 2C).

**Figure 3:**
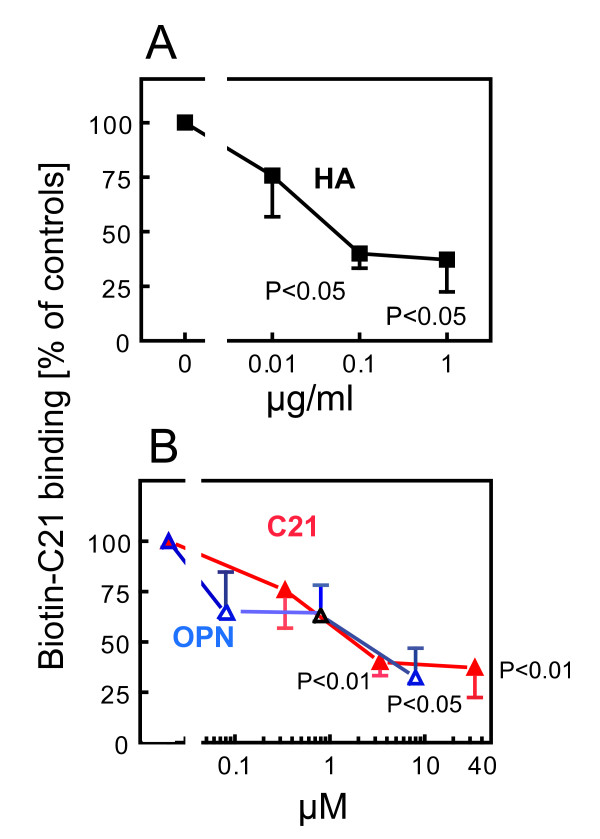
**HA and OPN compete for Biotin-C21 binding to CD44**. **(A) **Binding of Biotin-C21 to CD44 immobilized on nitrocellulose membranes was displaced with increasing concentrations of HA. No displacement is considered as 100% (controls). Mean ± SEM. n = 7. **(B) **Competition for Biotin-C21 binding to CD44 with increasing concentrations of C21 (closed triangles) or OPN (open triangles). Mean ± SEM. n = 5 for 8 μM OPN, n = 6 for 0.08 μM OPN and 0.33 μM C21, n = 8 for 0.8 μM OPN, n = 9 for 3.3 and 33 μM C21. P values calculated by analysis of variance and Student-Newman-Keuls test (C21 and HA) or Krustal-Wallis and Dunn's multiple comparisons test (OPN).

This protein was utilized to study the possible competition of biotin-C21 binding to CD44 by HA (Figure 3A) and OPN (Figure 3B). Both HA and OPN displaced Biotin-C21 binding to CD44. Surprisingly, the inhibition of Biotin-C21 binding caused by increasing amounts of C21 and OPN was practically identical (Figure 3B), suggesting common binding sites for the TSP-4 peptide and OPN. A similar comparison of C21 with HA was not possible, due to the undefined, heterogeneous molecular weight of the HA preparation.

### Opposite effects of C21 and OPN on colony formation of CD133+ cord blood cells

**Figure 4:**
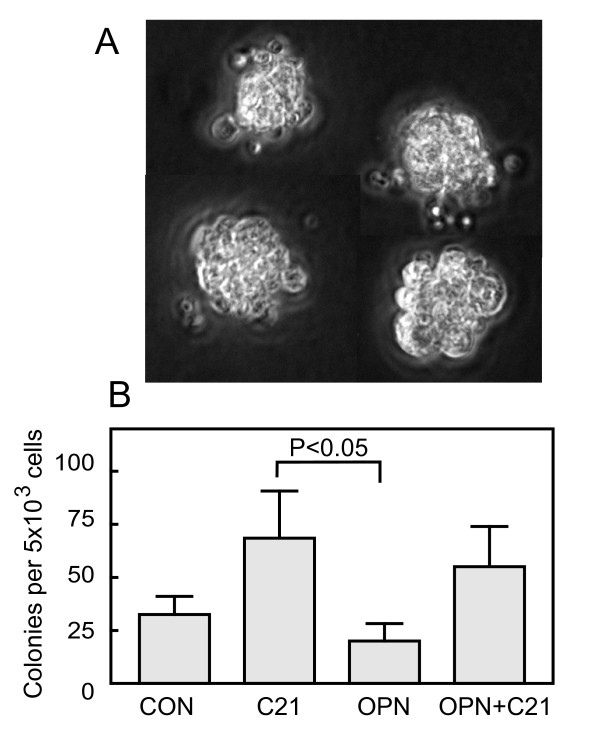
**Colony formation of CD133+ cord blood cells in the presence of C21 and OPN**. **(A) **Microscope images of 4 typical colonies formed after 18 days in culture of CD133+ cells in the semisolid serum-free medium described in Methods. The "Methocult"-medium contained stem cell factor, GM-CSF, G-CSF, IL-3, IL-6 and EPO. The culture was not done on stromal cell monolayers and did not contain Flt3 ligand, thrombopoietin or VEGF. **(B) **Total colony cell count observed after 18 days incubation with C21, OPN or C21+OPN. Mean ± SEM. n = 4. P value according to Friedman Test (nonparametric repeated measures analysis of variance) and Dunn's multiple comparisons test.

The most important function of OPN in hematopoiesis seems to be the suppression of proliferation of primitive stem cells in the bone marrow niche, as evidenced with OPN-/- mice and in vitro colony formation assays [16,17]. The effect of OPN depends on the differentiation state of the stem cells. The inhibitory action is eliminated with the appearance of the cell marker CD38+ [17] and in highly differentiated, primary erythroblasts, OPN actually stimulates cell proliferation [22]. TSP-1 has a similar differentiation-dependent effect on cell proliferation [3,5]. The existence of opposite effects of OPN in primitive precursors and differentiated cells is just one example of the more generalized duality of OPN action. This is illustrated by its double action as pro-inflammatory or anti-inflamatory cytokine, observed under normal or pathological conditions, as recently reviewed by Wang and Denhardt [23]. This multiplicity of biological activities, receptors and signal transduction pathways requires a careful choice in the nature and differentiation stage of the target cells to be used in the experiments. OPN-dependent inhibition by C21 has to be studied in very primitive, CD38- stem cell precursors. We chose CD133+ cells, which can differentiate into hematopoietic or endothelial cells [24,25]. For the experiments on colony formation, we used a minimal serum-free cell culture system without Flt3 ligand, thrombopoietin, VEGF or stromal cell monolayers, usually required for full differentiation into granulocyte-macrophage colonies, erythroid colonies or endothelial cells. Therefore, the colonies formed were compact and small (Figure 4A). Figure 4B shows that C21 and OPN had opposite effects on colony formation. This antagonistic action was significant. Although the OPN-mediated reduction of colony formation was not significantly different from that observed in control cell cultures in CD133+ cells, probably due to the low number of experiments, the inhibitory action of OPN in similar, primitive hematopoietic stem cells cultures is well established [16,17]. There is a correlation between the antagonistic effects of OPN and C21 on colony formation in vitro (Figure 4) with the observed molecular competition for CD44 binding in silico (Figure 3). The contribution of CD44 to this antagonism may be limited, because C21 could stimulate proliferation by CD44-independent pathways and OPN action on colony formation seems to be mediated by β1 integrins rather than CD44 [17]. A CD44 involvement is nevertheless quite possible. CD44 binding could passively increase the local, membrane bound concentration of OPN. The most probable scenario is that CD44 actively participates as a coreceptor, bound in lipid rafts to integrin chains. This association leads to integrin activation, as demonstrated in adenocarcinoma cell lines [26].

In conclusion, C21 could act on target cells by multiple mechanisms, which may involve competition with OPN for CD44 binding sites, ROD1-dependent signal transduction pathways involving proliferation and, probably, ROD1- independent effects on apoptosis, as previously discussed in [5].

## Competing interests

The authors declare that they have no competing interests.

## Authors' contributions

GS performed the experiments on affinity chromatography. GS and MCD did all experiments on plasmid construction, transduction, transfection, cell culture, binding studies and isolation of CD44. LFC wrote the original draft of the manuscript, performed the colony assays and participated in the purification of CD44. All authors read and approved the manuscript.
